# Sex Differences in High Sensitivity C-Reactive Protein in Subjects with Risk Factors of Metabolic Syndrome

**DOI:** 10.5935/abc.20160027

**Published:** 2016-03

**Authors:** Vinicius Pacheco Garcia, Helena Naly Miguens Rocha, Allan Robson Kluser Sales, Natália Galito Rocha, Antonio Claudio Lucas da Nóbrega

**Affiliations:** 1 Laboratório de Ciências do Exercício - Departamento de Fisiologia e Farmacologia - Universidade Federal Fluminense, Niterói, RJ - Brazil; 2 Unidade de Reabilitação Cardiovascular e Fisiologia do Exercício - Instituto do Coração (InCor) - Faculdade de Medicina da Universidade de São Paulo, São Paulo, SP - Brazil

**Keywords:** Metabolic Syndrome, Risk Factors, Sex Characteristics, Protein C

## Abstract

**Background:**

Metabolic syndrome (MetS) is associated with a higher risk of all-cause mortality. High-sensitivity C-reactive protein (hsCRP) is a prototypic marker of inflammation usually increased in MetS. Women with MetS-related diseases present higher hsCRP levels than men with MetS-related diseases, suggesting sex differences in inflammatory markers. However, it is unclear whether serum hsCRP levels are already increased in men and/or women with MetS risk factors and without overt diseases or under pharmacological treatment.

**Objective:**

To determine the impact of the number of MetS risk factors on serum hsCRP levels in women and men.

**Methods:**

One hundred and eighteen subjects (70 men and 48 women; 36 ± 1 years) were divided into four groups according to the number of MetS risk factors: healthy group (CT; no risk factors), MetS ≤ 2, MetS = 3, and MetS ≥ 4. Blood was drawn after 12 hours of fasting for measurement of biochemical variables and hsCRP levels, which were determined by immunoturbidimetric assay.

**Results:**

The groups with MetS risk factors presented higher serum hsCRP levels when compared with the CT group (p < 0.02). There were no differences in hsCRP levels among groups with MetS risk factors (p > 0.05). The best linear regression model to explain the association between MetS risk factors and hsCRP levels included waist circumference and HDL cholesterol (r = 0.40, p < 0.01). Women with MetS risk factors presented higher hsCRP levels when compared with men (p_sex_ < 0.01).

**Conclusions:**

Despite the absence of overt diseases and pharmacological treatment, subjects with MetS risk factors already presented increased hsCRP levels, which were significantly higher in women than men at similar conditions.

## Introduction

Metabolic syndrome (MetS) is a cluster of metabolic risk factors that includes high blood pressure, hyperglycemia, dyslipidemia, and abdominal obesity. When these risk factors are present together, the probability of future cardiovascular problems becomes greater than with any of the factors alone.^1,2^ Previous studies estimate that 40% of North Americans^3^ and 25% of Europeans^4^ or Latin Americans^5^ may present MetS by the time they reach the age of 60 years. Currently, most efforts are directed towards early detection and treatment of individuals with established MetS to avoid the development of cardiovascular disease.

Patients with MetS usually present increased levels of high-sensitivity C-reactive protein (hsCRP), which is a prototypic marker of inflammation.^6^ Several studies have shown that there is a clear relationship between metabolic disorders and higher hsCRP levels.^7,8^ It has also been shown that women with cardiometabolic risks, *i.e.* those with MetS, diabetes, or hypertension, usually present higher hsCRP levels than men with MetS-related diseases, suggesting sex differences in inflammatory markers.^9^ However, it is unclear whether serum hsCRP levels are already increased in subjects with MetS risk factors and without overt diseases or under pharmacological treatment. Also, information about hsCRP levels in men and women with MetS risk factors are inconclusive.

Cardiometabolic diseases seem to have a cumulative effect on serum hsCRP levels. It has been demonstrated that hsCRP levels are higher in patients presenting simultaneously MetS and type 2 diabetes than in those with MetS alone.^10^ However, it is unknown whether the number of MetS risk factors (two or less, three, or four or more factors) can influence the levels of serum hsCRP. Considering these aspects together, this study aimed to determine the effects of the number of MetS risk factors on serum hsCRP levels in women and men. We hypothesized that serum hsCRP levels would increase according to the number of MetS risk factors in women.

## Methods

### Ethical approval

The study protocol was approved by the ethics committee of Fluminense Federal University and conformed to the standards set by the latest revision of the Declaration of Helsinki. All subjects gave written informed consent before participating in the study.

### Sample

Subjects were recruited through advertisements at the University and in local newspapers. One hundred and eighteen subjects (70 men and 48 women) aged 36 ± 1 years were enrolled. We considered the following risk factors of MetS:^11^ 1) waist circumference ≥ 90 cm in men and ≥ 80 in women; 2) serum triglycerides levels ≥ 150 mg/dL; 3) serum HDL cholesterol levels < 40 mg/dL in men and < 50 mg/dL in women; 4) systolic blood pressure ≥ 130 mmHg and/or diastolic blood pressure ≥ 85 mmHg; and 5) fasting serum glucose levels ≥ 100 mg/dL. We divided the subjects into four groups according to the number of MetS risk factors: healthy group (CT; no risk factors); MetS ≤ 2 (two or fewer risk factors); MetS = 3 (three risk factors); and MetS ≥ 4 (four or more risk factors). Other inclusion criteria included the absence of any diagnosed disease, recent infection, use of medication (except contraceptives) or smoking, and the presence of regular menstrual cycles (in women) and sedentary lifestyle (defined as lack of engagement in exercise activities lasting ≥ 30 min, three times per week during the last 3 months).

### Measurements

The subjects visited the laboratory three times. On the first visit, a physician conducted an evaluation that included assessment of clinical history and resting electrocardiogram (CardioCare 2000, Bionet, Tustin, CA, USA). On the second visit, the patients underwent a physical evaluation. Anthropometric variables, such as weight and height, were measured using a calibrated medical beam scale (Welmy, Santa Bárbara d´Oeste, SP, Brazil). Body mass index (BMI) was calculated as weight (in kilograms) divided by the squared height (in meters). Waist circumference was measured at the midpoint between the iliac crest and the lower (XII) rib. Blood pressure was measured twice, once in each arm, on two separate days (at the first and second visits) and with the patient in the upright sitting position. On the third visit, blood was drawn from the subjects.

### Biochemical blood analyses and hsCRP

Blood was drawn from an anterior cubital vein in the morning after a 12-hour fast. Cholesterol and its subfractions (HDL cholesterol, low-density lipoprotein [LDL] cholesterol, and very-low-density lipoprotein [VLDL] cholesterol) as well as triglycerides and glucose were determined using enzymatic colorimetric methods. Serum levels of hsCRP were measured by immunoturbidimetric assay (Tina-quant® latex, Roche, Basel, Switzerland).

### Statistical methods

The data distribution was assessed by the Shapiro-Wilk test. A total sample size of 110 subjects was necessary to detect differences on CRP concentration among the groups (group main effect), considering a one-way ANOVA p value of 0.05 and power of 0.90. Two-way repeated measures ANOVA was also used to compare the variable hsCRP among MetS risk factors groups between males and females. Associations between hsCRP and individual components of MetS were determined by multiple linear regression analysis. Data are presented as mean ± standard error of the mean (SEM). Significance was accepted at a 0.05 level. All analyses were performed with the software Statistica (version 8, StatSoft Inc., Oklahoma, USA).

## Results

Table 1 presents the anthropometric, metabolic, and hemodynamic variables. The groups matched for sex and age (p > 0.05). Waist circumference, BMI, VLDL cholesterol, and triglycerides were significantly different in the CT groups compared with the MetS risk factors groups. In addition, the MetS = 3 and MetS ≥ 4 groups also had higher BMI, waist circumference, systolic blood pressure, and serum levels of VLDL cholesterol, triglycerides, and glucose, as well as lower serum HDL cholesterol levels compared with the MetS ≤ 2 group (p < 0.05).

**Table 1:** Biochemical and hemodynamic characteristics of healthy subjects and individuals with MetS risk factors

**Variables**	**Groups**			
	**CT**	**MetS ≤ 2**	**MetS = 3**	**MetS ≥ 4**
N (M/W)	18 (11/7)	67 (34/33)	23 (19/4)	10 (6/4)
Age (years)	33 ± 2	36 ± 1	37 ± 1	39 ± 1
BMI (kg/m^2^)	22.90 ± 0.62	28.76 ± 0.40*	32.26 ± 0.88*†	31.15 ± 0.97*†
Waist circumference (cm)	78.98 ± 1.88	95.02 ± 1.12*	105.86 ± 1.70*†	103.2 ±1.54*†
SBP (mmHg)	114 ± 1	116 ± 1	126 ± 3*†	129 ± 1*†
DBP (mmHg)	75 ± 1	76 ± 1	81 ± 2	85 ± 3*†
Total cholesterol (mg/dL)	174.89 ± 6.77	193.30 ± 4.94	207.22 ± 8.53*	215.30 ± 8.66*
HDL-c (mg/dL)	56.67 ± 2.60	53.85 ± 1.47	41.48 ± 1.54*†	38 ± 2.82*†
LDL-c (mg/dL)	102.06 ± 6.68	119.77 ± 4.50	124 ± 7.63	138.38 ± 8.43*
VLDL-c (mg/dL)	13.11 ± 1.25	19.56 ± 0.85*	41.87 ± 3.24*†	46.13 ± 4.42*†
Triglycerides (mg/dL)	54.83 ± 2.35	98.52 ± 4.51*	209.09 ± 16.28*†	230.88 ± 22.26*†
Glucose (mg/dL)	86.72 ± 1.57	87.61 ± 0.76	95.76 ± 2.39*†	97 ± 4.40*†

Values are displayed as mean ± SEM. CT: healthy subjects; MeTS: metabolic syndrome; BMI: body mass index; SBP: systolic blood pressure; DBP: diastolic blood pressure; LDL-c, low-density lipoprotein cholesterol; HDL-c, high-density lipoprotein cholesterol.

(*) p < 0.05 vs. CT;

(†) p < 0.05 vs. MetS ≤ 2.

**Figure 1:**
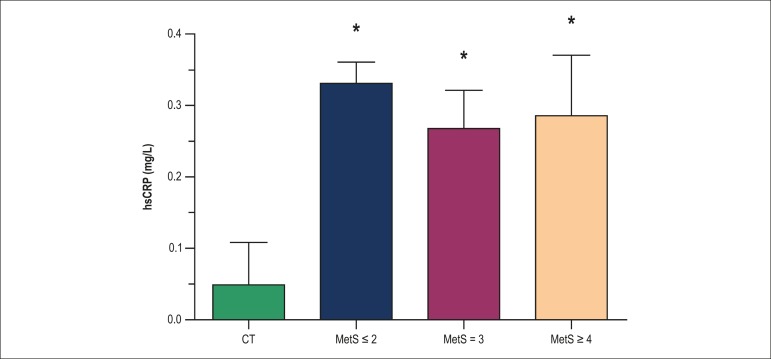
Distribution of serum hsCRP levels according to number of MetS risk factors. (*) p < 0.05 vs. CT.

The groups with MetS risk factors presented higher serum hsCRP levels compared with the CT group (p ≤ 0.02). However, there were no differences in hsCRP levels among groups with MetS risk factors (Figure 1; p > 0.05). When the analysis was adjusted for BMI, similar results were noted (data not shown).

Stepwise multivariate regression analysis of serum hsCRP levels and MetS risk factors demonstrated that waist circumference and HDL cholesterol levels were the major predictors of increased hsCRP levels [y = -1.214+0.13*(waist circumference)+ 0.0006*(HDL cholesterol)] (r = 0.40, p < 0.01).

**Figure 2:**
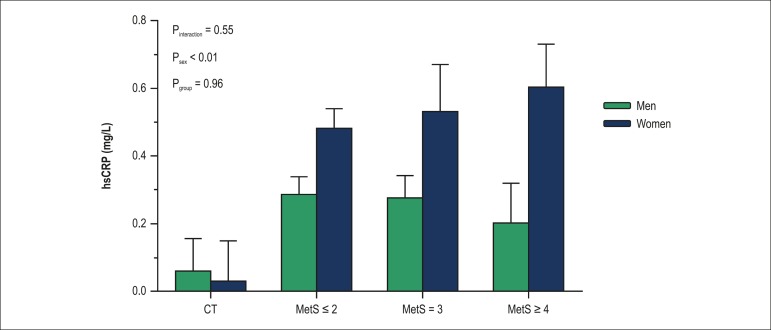
- Serum hsCRP levels in men and women according to the number of MetS risk factors. hsCRP: high-sensitivity C-reactive protein; CT: healthy subjects; MetS: metabolic syndrome; MetS ≤ 2, subjects with one or two MetS risk factors; MetS = 3, subjects with three MetS risk factors; MetS ≥ 4, subjects with four or five MetS risk factors.

Regarding sex-differences on hsCRP levels, no difference was observed between women and men in the CT group (p = 0.84), whereas in the groups with MetS- related risk factors, women presented higher levels of hsCRP when compared with men (p_sex_ < 0.01). However, hsCRP levels were still similar among the groups with MetS risk factors (p > 0.05) (Figure 2).

## Discussion

In this study, we tested the hypothesis that serum levels of hsCRP would increase according to the number of MetS risk factors in women. New findings of our study were threefold: 1) hsCRP levels were already higher in subjects with MetS risk factors when compared with controls; 2) the number of MetS risk factors did not influence the levels of hsCRP; 3) women with MetS risk factors presented higher hsCRP levels when compared with men with MetS risk factors.

Previous studies have shown associations between markers of inflammation and components of MetS.^12,13^ CRP levels in subjects with MetS have also been reported to be four times higher than those in healthy subjects.^12^ Our study demonstrated that subjects with MetS risk factors, even without preexisting diseases or under pharmacological treatment, already present early changes in hsCRP levels. Taken together, these data suggest that MetS risk factors may be associated with systemic low-grade inflammation.

We found no differences in hsCRP levels among groups with MetS risk factors. In contrast, other studies have demonstrated that CRP levels are positively associated with the number of MetS components.^7,14^ These studies have also demonstrated a gradual increase in CRP levels with the number of MetS components. It is important to observe that subjects in these other studies presented overt cardiometabolic diseases and/or were taking regular medications. Thus, increased serum CRP levels could be associated with the number of cardiometabolic diseases.

Waist circumference and HDL colesterol levels were the best predictors to explain the increase in hsCRP levels in subjects with MetS risk factors. Nakamura et al. have shown that among the MetS components, waist circumference is the main determinant of increase in CRP concentrations.^15^ Several studies have reported an inverse relationship between levels of HDL cholesterol and CRP in healthy individuals and subjects with MetS, suggesting that low HDL cholesterol levels may favor the inflammatory process.^16,17^

In a previous study from our group, we have shown that subjects with MetS, even without overt diseases or under pharmacological treatment, already present an early endothelial dysfunction, demonstrated by a longer time to peak diameter and an increased sE-selectin level.^18^ Endothelial dysfunction appears to stimulate an inappropriate secretion of proinflammatory and anti-inflammatory adipocytokines in subjects with MetS^19^ and may lead to a systemic inflammatory condition, which activates genes encoding CRP and other agents in the acute phase.

Regarding sex differences on hsCRP levels in groups with MetS risk factors, women presented higher levels of hsCRP when compared with men. Han et al. also demonstrated that CRP levels predict the development of MetS in women but not in men.^20^ The sex differences observed in these studies could be explained by endogenous synthesis of estrogen, a hormone that might play a role on the inflammatory process in women. An alternative explanation would be that women might have a greater amount of total body adipose tissue compared with men, which could be the source of proinflammatory cytokines.^20^

We must mention a limitation of our study. Values of BMI were different among the CT and MetS risk factors groups (MetS ≤ 2, MetS = 3, and MetS ≥ 4). This is important, since it is well known that obesity *per se* induces an inflammatory response and increases the serum levels of hsCRP.^13^ However, we obtained similar results when we performed a BMI-adjusted analysis.

## Conclusion

Despite the absence of overt diseases and pharmacological treatment, subjects with MetS risk factors presented increased hsCRP levels when compared with healthy subjects. Waist circumference and HDL cholesterol were identified as independent predictors of increased serum hsCRP levels in subjects with MetS risk factors. Moreover, women with MetS risk factors presented higher hsCRP levels than men in the same condition.

These results indicate that an unspecific and subclinical inflammatory process is already present in early stages of the natural history of MetS, through the presence of high hsCRP levels. Measurements of this acute phase inflammatory protein may help determine an individual's cardiovascular risk and implement effective preventive strategies to avoid the development of cardiometabolic diseases, mainly in women.
